# Application of Cellular Technologies to the Experimental Treatment of Destructive Inflammatory Arthropathies

**DOI:** 10.5195/cajgh.2014.161

**Published:** 2014-12-12

**Authors:** Marzhan Kaulambayeva, Galina Fedotovskikh, Anara Nurmukhambetova

**Affiliations:** 1 RPE Antigen Ltd, Kazakhstan; 2 National Research Medical Center, Astana, Kazakhstan

**Keywords:** arthropathies, mononuclear cells, mesenchymal stromal cells, joint diseases

## Abstract

**Introduction:**

The treatment of destructive inflammatory joint diseases (arthropathies) is one of the issues of current interest in modern medicine. In destructive inflammatory diseases, the regenerative ability of cartilaginous tissue proves to be inadequate for neogenesis of joints. The goal of this study is to determine the efficacy of bone marrow-derived mononuclear cell fraction (MNC) and multipotent mesenchymal stromal cells (MMSC) in the treatment of destructive inflammatory joint diseases.

**Materials and methods:**

The study subjects consisted of 15 male rabbits weighing 3–4 kg with experimental destructive inflammatory knee joint disease. The test animals were divided into 3 groups: reference group without treatment, first test group – introduction of autologous MNC from rabbit bone marrow into the affected joint, and second test group – introduction of cultured MMSC from rabbit bone marrow into the joint.

**Results:**

A morphological examination of the synovial membranes in the reference group on the 40th day of the experiment revealed chronic synovitis with destruction of synoviocytes, thickening and inflammatory infiltration of the underlying connective tissue (subintima). During examination of synovial membranes in the first test group the patches of thickened regenerating inner layer (intima) made up by large proliferating synoviocytes were observed. The layer of loose connective tissue (subintima) contained a large number of small blood vessels and was only slightly infiltrated by inflammatory cells. The morphological examination of synovial membranes in the second test group discovered thickened regenerating intimal layer sitting on hypertrophied subintima with dense vascular network. Elastic collagenous layers of synovial membrane adjoined proliferating elements in cartilage plates.

**Conclusion:**

Both autologous MNC fraction and MMSC from bone marrow proved effectiveness in the treatment of destructive inflammatory joint diseases which stimulate neoangiogenesis. At the same time, it must be noted that the introduction of MMSC diminished destructive changes and accelerated proliferative process.

